# Detecting depression of Chinese microblog users *via* text analysis: Combining Linguistic Inquiry Word Count (LIWC) with culture and suicide related lexicons

**DOI:** 10.3389/fpsyt.2023.1121583

**Published:** 2023-02-09

**Authors:** Sihua Lyu, Xiaopeng Ren, Yihua Du, Nan Zhao

**Affiliations:** ^1^CAS Key Laboratory of Behavioral Science, Institute of Psychology, Chinese Academy of Sciences, Beijing, China; ^2^Department of Psychology, University of Chinese Academy of Sciences, Beijing, China; ^3^Computer Network Information Center, Chinese Academy of Sciences, Beijing, China

**Keywords:** CES-D, depression, prediction, microblogging, machine learning, text mining

## Abstract

**Introduction:**

In recent years, research has used psycholinguistic features in public discourse, networking behaviors on social media and profile information to train models for depression detection. However, the most widely adopted approach for the extraction of psycholinguistic features is to use the Linguistic Inquiry Word Count (LIWC) dictionary and various affective lexicons. Other features related to cultural factors and suicide risk have not been explored. Moreover, the use of social networking behavioral features and profile features would limit the generalizability of the model. Therefore, our study aimed at building a prediction model of depression for text-only social media data through a wider range of possible linguistic features related to depression, and illuminate the relationship between linguistic expression and depression.

**Methods:**

We collected 789 users’ depression scores as well as their past posts on Weibo, and extracted a total of 117 lexical features *via* Simplified Chinese Linguistic Inquiry Word Count, Chinese Suicide Dictionary, Chinese Version of Moral Foundations Dictionary, Chinese Version of Moral Motivation Dictionary, and Chinese Individualism/Collectivism Dictionary.

**Results:**

Results showed that all the dictionaries contributed to the prediction. The best performing model occurred with linear regression, with the Pearson correlation coefficient between predicted values and self-reported values was 0.33, the R-squared was 0.10, and the split-half reliability was 0.75.

**Discussion:**

This study did not only develop a predictive model applicable to text-only social media data, but also demonstrated the importance taking cultural psychological factors and suicide related expressions into consideration in the calculation of word frequency. Our research provided a more comprehensive understanding of how lexicons related to cultural psychology and suicide risk were associated with depression, and could contribute to the recognition of depression.

## 1. Introduction

Depression has been considered as a common mental illness worldwide, affecting an estimated 3.8% of the population, including 5.0% of young adults and 5.7% of adults over 60 (1). Globally, approximately 280 million people suffered from depression (1). In China, it was estimated nearly 50 million people had depression, accounting for 3.6% of the country’s population (1). In addition, studies have shown that the prevalence of depressive symptoms among different age groups and occupational groups ranged from 17 to 40% (2–6).

Depression has a significantly negative effect on patients, leading to poorer quality of life, cognitive dysfunction, low work productivity and unemployment (7–10). Moreover, it also poses a huge economic and psychological burden for both the family and society (11–14). Recent research further suggested that COVID-19 pandemic has increased the prevalence and burden of depressive disorders, especially for some vulnerable populations such as females, younger people and medical staff (15–17). As the early detection and intervention of depression could mitigate negative effects associated with depression (18, 19), it is of great value to provide depression screening and tracking services, especially for those vulnerable groups.

Many depression assessment scales such as Center for Epidemiological Studies Depression Scale (CES-D) have been designed for depression screening. And U.S. Preventive Services Task Force (20) demonstrated these scales have good sensitivity (80–90%) and fair specificity (70–85%). Nevertheless, two major disadvantages will arise when we use these scales for routinely depression screening at large scale. Firstly, although online technology has eased the difficulty and cost of conducting traditional questionnaires, the survey response rate still needs to be improved (21, 22), especially the follow-up surveys. Secondly, large-scale depression screening can be time-consuming to collect data from the targeted population.

Given these shortcomings, increasing number of studies have investigated the possibility of screening depressive symptoms passively and automatically. Some researchers have showed the potential of using neurophysiological biomarkers and text data collected from social media platform to detect and measure depression (23–25). With the development of the online technology, it is obviously more cost-effective to screen depression in large-scale through social media. And it is of our concern how to understand and improve the prediction models of depression using social media data.

Throughout the literature, previous research based on social media platform has mainly made use of three types of features while training the model, that is, social networking behaviors, profile features, and psycholinguistic features (26–31). In terms of psycholinguistic features, it could be found that the most widely adopted approach for feature extraction is to use the Linguistic Inquiry Word Count (LIWC) dictionary and various affective lexicons. Other factors in individual characteristics have not been included in the analysis of textual content in past research.

However, sociocultural factors have been considered by increasing number of mental health scholars and practitioners in their conceptions of causality and treatment of mental disorders. For example, Marsella et al. (32) proposed the interactional model of behavior, in which relating both physical environment and socioenvironmental phenomena to individuals’ biological and psychological variables. Besides, many studies have explained how culture-related factors were entangled with individuals’ mental health. Specifically, Helgeson (33) stated that moral motivation such as communion could be beneficial to mental well-being through improving relationship satisfaction. Moreover, Kirsh and Kuiper (34) pointed out that people with excessive individualism and relatedness were more likely to engage in the negative kind of thinking. Furthermore, the discrepancies between ones’ own moral standards and those of society could positively predict depression (35). Thus, cultural factors such as moral motivation, individualism, and moral foundation might have an impact on mental health, in particular depression. Moreover, Coryell and Young (36) found depressive individuals would have higher suicide risk. Thus, it also seems plausible to use suicidal expression to help train the detection model.

Checking wider ranges of possible linguistic features would introduce a more comprehensive understanding of the relationship between real-life verbal expression and depression, and also might increase the performance of the prediction model. Furthermore, developing and optimizing the linguistic features would do great help to model generalization, as the use of social networking behavioral features and profile features usually make it impossible to apply the model in different platform or text-only condition.

Therefore, our study aimed at building a prediction model of depression for text-only social media data so that the model could be generalized across platforms. Furthermore, wider ranges of depression-related lexicons were tested in order to explore how these lexical features entangled with depression. Specifically, not only did we use Simplified Chinese Linguistic Inquiry Word Count (SCLIWC) dictionary, but also covered psycholinguistic features representing suicidal expression, moral foundation, moral motivation and individualism/collectivism.

## 2. Materials and methods

### 2.1. Participants

We recruited 1,813 Chinese users of Sina Weibo, the most popular Chinese social media, to participate in this study. Participants were informed about the research and required to fill in the electronic consent form. Referring to the participant screening procedures of prior research (37–39), the inclusion criteria of participants for our study were as follows: (1) users who had over 50 posts with a total word count of more than 500 words in the month before the questionnaire was completed (*n* = 895); (2) at least 70% of the words should be identifiable by SCLIWC lexicon (*n* = 851); (3) users whose questionnaire completion time was over 90 s (*n* = 789). Finally, 789 valid participants were left (287 males), averaged 24.3 years of age (SD = 6.2, range = 13–57).

### 2.2. Measurement

#### 2.2.1. CES-D

The CES-D scale was used in the study to assess self-reported depressive symptoms (40). The CES-D consists of 20 items and all the statements could be rated on a scale of 0–3, with 0 representing rarely no symptom presence (less than 1 day in the past week) and 3 indicating most or all of the time (5–7 days in the past week). Previous research has suggested cutoff scores to differentiate patients with different depressive symptoms severity: (1) no depression, score 0–15; (2) mild depression, score 16–20; (3) moderate depression, score 21–25; (4) severe depression, score 26–60 (40, 41).

### 2.3. Procedure

#### 2.3.1. Data collection

**FIGURE 1:**
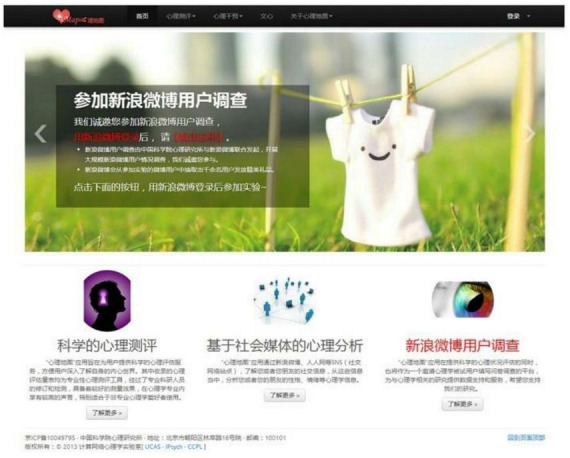
“XinLiDiTu” website.

We developed a Weibo-based application named “XinLiDiTu” to recruit participants (Figure 1). Weibo users could get paid by filling out an online survey containing CES-D scale and demographic questions (e.g., gender, age). Then, a crawler would collect their original public Weibo posts from the pre-constructed Weibo data pool (42). It should be noted that we only collected the posts in the month prior to the day the survey was completed, as the CES-D scale was designed to measure current psychological state.

#### 2.3.2. Feature extraction

We employed the Simplified Chinese LIWC (SCLIWC) dictionary, Chinese Suicide Dictionary, Chinese Version of Moral Foundations Dictionary, Chinese Version of Moral Motivation Dictionary and Chinese Individualism/Collectivism Dictionary to extract word frequency features from Weibo posts.

**SCLIWC** mapped the written expression into over 80 psychologically or linguistically meaningful categories, covering individuals’ psychological aspects such as emotional and cognitive processes (43).

**Chinese Suicide Dictionary** consists of 2,168 words, which belongs to 13 different categories related to the risk of suicide (e.g., suicide ideation) (44).

**Chinese Version of Moral Foundations Dictionary** is composed of 580 words for five moral foundations (Care/Fairness/Loyalty/Authority/Sanctity), with each foundation containing both foundation-supporting words (virtues) and foundation-violating words (vices) (45, 46).

**Chinese Version of Moral Motivation Dictionary** was adapted by Zhang and Yu (47) from the work of Frimer (48). The Chinese version one has 690 words for the agency dimension and 260 words for the communion dimension.

**Chinese Individualism/Collectivism Dictionary** was developed by Ren et al. (49), including 53 individualism words and 64 collectivism words.

We aggregated all the posts of each individual into a single text and calculate the word frequency of lexical categories from above dictionaries. Finally, a total of 117 lexical features were extracted from our dataset.

#### 2.3.3. Feature selection

**FIGURE 2:**
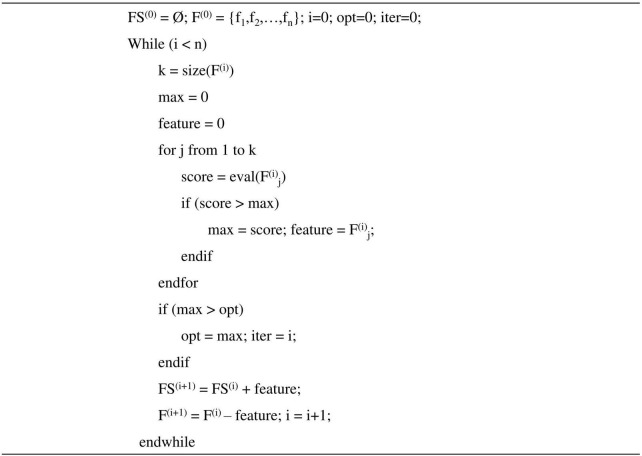
Forward greedy feature selection. FS^(i)^ represents the selected features in i-th iteration, F^(i)^ represents features that have not been selected in i-th iteration.

To optimize the performance of regression models, the greedy algorithm was adopted for search strategy in the feature selection. The greedy stepwise forward algorithm would add one feature per step that provides the highest increase in evaluation measure. And it will terminate when the evaluation measure is not improved, or the variables run out. The whole process was described in Figure 2. In our study, the Pearson correlation coefficients between predicted values and true values were used as the evaluation measure.

## 3. Results

### 3.1. Descriptive statistics

The scores of the CES-D scale were distributed over a relatively wide range from 0 to 57, with a mean of 17.15 and a standard deviation of 11.67. Table 1 shows the percentage distribution of no depression and depression with different level of symptoms [i.e., mild, moderate, severe; (40, 41)] among all subjects. It can be seen that near half of the sample had depressive symptomatology to some extent.

**TABLE 1:** The percentage distribution of subjects with different level of depressive symptoms.

Depression level	Number of subjects	Percentage
No depression	408	51.71%
Mild depression	117	14.83%
Moderate depression	96	12.17%
Severe depression	168	21.29%

### 3.2. Correlation between lexical features and depression

**FIGURE 3:**
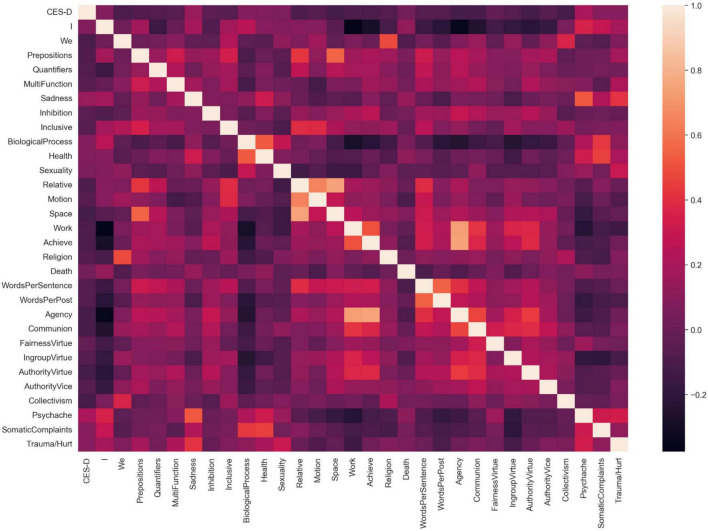
The heatmap between lexical features and CES-D score. *I, We, Prepositions, Quantifiers, MultiFunction, Sadness, Inhibition, Inclusive, BiologicalProcess, Health, Sexuality, Relative, Motion, Space, Work, Achieve, Religion, Death, WordsPerSentence*, and *WordsPerPost* were extracted from SCLIWC; *Agency and Communion* were extracted from The Chinese Version of Moral Motivation Dictionary; *Fairness Virtue, IngroupVirtue, AuthorityVirtue, AuthorityVice* were extracted from The Chinese Version of Moral Foundation Dictionary, *Collectivism* was extracted from The Chinese Individualism/Collectivism Dictionary; *Psychache, SomaticComplaints*, and *Trauma/Hurt* were extracted from The Chinese Suicide Dictionary.

We calculated Spearman correlations coefficients between word frequency features and CES-D scores. Before Bonferroni correction, a total of 30 lexical features that were significantly correlated with depression were eventually obtained from these five dictionaries (see Figure 3). Among them, psychache words from Chinese Suicide Dictionary reached the highest correlation coefficient with depression scores (*r* = 0.19, *p* < 0.001). After performing Bonferroni correction, only space words (*r* = −0.13, *p* < 0.001) from SCLIWC and psychache words (*r* = 0.19, *p* < 0.001) significantly correlated with CES-D scores.

### 3.3. The performance of regression models

To evaluate the performance of regression models, we adopted 5-fold cross-validation to calculate the mean of R-squared scores and the mean of Pearson correlation coefficients between predicted values and true values for each algorithm. We used ridge, linear regression, support vector regression, random forest regression, and gradient boosting regression to build machine learning models. To validate the effectiveness of the culture and suicide related lexicons in predicting depression, we compared the results for data with and without these added features (full dataset vs. SCLIWC dataset). The top three best performing models for both datasets have been illustrated in Table 2, supporting that the performance of the predictive model could be improved with culture and suicide related features.

**TABLE 2:** The performance of the regression models with 5-fold-cross validation.

Dataset	Method	*R* ^2^	*r*
SCLIWC + ^1^culture related lexicons + ^2^suicide related lexicons	Linear regression	0.10	0.33
	Support vector regression	0.02	0.25
	Random forest regression	0.02	0.20
SCLIWC	Linear regression	0.04	0.26
	Gradient boosting regression	0.03	0.24
	Random forest regression	0.03	0.23

^1^Culture related lexicons included features extracted from Chinese Version of Moral Foundations Dictionary, Chinese Version of Moral Motivation Dictionary, and Chinese Individualism/Collectivism Dictionary; ^2^suicide related lexicons included features extracted from Chinese Suicide Dictionary.

**FIGURE 4:**
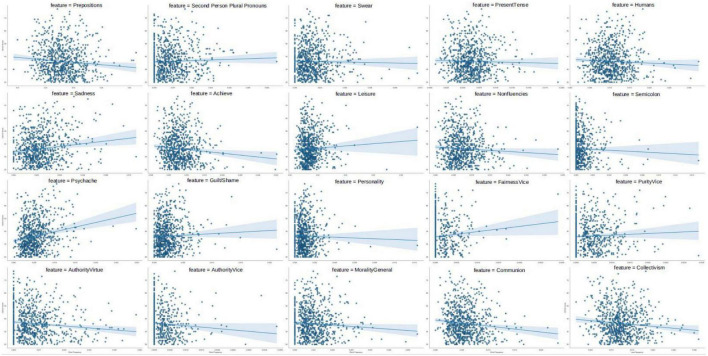
Scatter plots of selected features with CES-D scores. The horizontal axis represents the word frequency, and the vertical axis represents the CES-D score.

For the full dataset, the best outcome occurred with linear regression (*R*^2^ = 0.10, *r* = 0.33), with the selected features shown in Table 3. And the scatterplot of these selected features with CES-D scores presented in Figure 4. It can be seen that some of the features (e.g., psychache) significantly associated with depression scores were also selected into the model.

**TABLE 3:** Selected features after feature selection.

Dictionary	Features
SCLIWC	Prepositions Second person plural pronouns Swear Present tense Humans Sadness Achieve Leisure Non-fluencies Semicolon
Chinese Suicide Dictionary	Psychache Guilt/shame Personality
Chinese Version of Moral Foundations Dictionary	Fairness vice Purity vice Authority virtue Authority vice Morality general
Chinese Version of Moral Motivation Dictionary	Communion
Chinese Individualism/Collectivism Dictionary	Collectivism

### 3.4. The split-half reliability of regression models

**FIGURE 5:**
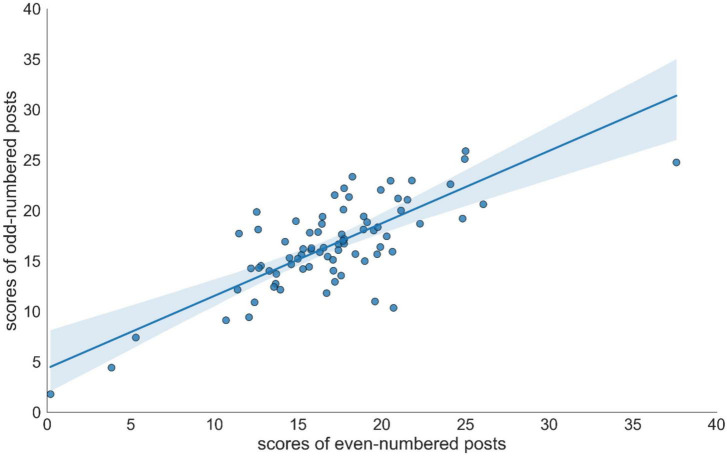
User’s depression scores calculated from even-numbered posts and odd-numbered posts.

To obtain the split-half reliability, we were supposed to split our samples into two parts, one of which was used for model building and the other for testing. Thus, we ranked users in descending order of the number of posts and selected the last 90% of users (*n* = 711) to build the CES-D model. While developing the model, we still used the greedy forward stepwise algorithm for feature selection and linear regression for model training. For the remaining top 10% (*n* = 78) of users, each individual’s Weibo posts were sorted by posting time and ordered by ascending. Further, their posts were divided into halves individually (i.e., odd-numbered and even-numbered posts). Then, the CES-D model predicted the scores for each individual based on the odd-numbered and the even-numbered posts, so there were two CES-D scores for each user. The scatterplot (Figure 5) shows a strong and positive relationship (*r* = 0.75, *p* < 0.01) between these two scores.

## 4. Discussion

### 4.1. The feasibility of recognizing depression *via* word frequency information

In this study, lexical features were extracted using SCLIWC, Chinese Suicide Dictionary, Chinese Version of Moral Foundations Dictionary, Chinese Version of Moral Motivation Dictionary and Chinese Individualism/Collectivism Dictionary to build predictive models of depression through machine learning methods. Results show that the correlation between actual scores and predicted scores achieved 0.33, and the split-half reliability was 0.75.

In fact, Hu et al. (50) has used a total of 927 features, including linguistic features (*via* SCLIWC), stable behavioral features (i.e., profile features, self-expression behaviors, privacy settings, interpersonal behaviors), and dynamic behavioral features (i.e., microblog updates, mentions, use of apps, recordable browsing behaviors) to build depression recognition model. The Pearson correlation coefficients between predicted values and self-reported values of the best-performance model in their study reached 0.38. Although their model slightly outperformed our model, they used more complex and not easily accessible data such as recordable browsing behavior. By taking cultural psychological factors and suicide expressions into consideration in the calculation of word frequency, we could obtain a model that was slightly inferior in performance, but has greatly reduced the difficulty and complexity of feature extraction. Moreover, using only lexical features could improve the generalization ability of the model, so that the application of the model could not only be limited to the microblogging platform.

Moreover, we can see that the linear regression offered the best outcome in terms of the model performance and split-half reliability. It might imply a linear relationship between word frequencies in public discourse and users’ depression. Compared to random forest regression and support vector machine, one of the advantages for linear regression models was that they were easier to interpret. By building a linear model, it provided new evidence for us to reveal and understand the expression pattern of depressive populations.

### 4.2. The features contributed in depression identification

Compared with correlation analysis, linear regression could estimate the change in the depression scores due to the change in one or more independent variables. Thus, the features selected by the linear regression model did contribute to the detection of depression, to varying degrees. Half of the chosen features were from SCLIWC: prepositions, second person plural pronouns, semicolon, present tense, leisure, achieve, humans, swear, non-fluencies and sadness. Another half of the selected features were from cultural psychological dictionaries and the suicide dictionary: fairness vice, purity vice, authority virtue, authority vice and morality general from Chinese Version of Moral Foundations Dictionary; communion from Chinese Version of Moral Motivation Dictionary; collectivism from Chinese Individualism/Collectivism Dictionary; psychache, guilt/shame and personality from Chinese Suicide Dictionary. In the following section, we would elucidate how some of the above selected features could entangle with depression.

#### 4.2.1. SCLIWC

Linguistic Inquiry Word Count has been widely used to investigate the linguistic markers of depressed people (26–29, 51). And the findings suggest that depressed patients differed in the writing pattern comparing to the non-depressed group. These differences enable us to detect depression through plain text. Our findings were partially consistent with the previous literature, while some cross-cultural differences emerged.

Firstly, different from most of the past studies (52–54), first person singular pronouns did not serve an important role in the model. Based on self-awareness theory, self-focused attention was one of the vulnerability factors for the onset and maintenance of depression (55, 56). Thus, the use of first singular pronouns has long been considered as an effective indication of depressive narratives. We considered this discrepancy could be explained by two reasons: firstly, the absence of the subject was very common in Weibo comments posted by depressed individuals (57); secondly, first person singular pronouns in Chinese did not merely refer to the addresser himself or herself, but also used as pragmatic empathetic deixis to narrow the psychological distance between addresser and addressee (58). Therefore, we suspected that more research is needed to support the use of first person singular pronouns as a significant indicator of depression in the Chinese corpus.

Secondly, we observed the second person plural pronouns appear more frequently in the depression group. This was the opposite of the results obtained from studies conducted in English texts (54). However, this result replicated the findings of studies focusing on Chinese texts (59, 60). This could possibly because the social media has become a platform for the depressed group to communicate for social support and advice (61, 62), and thus they would use the second person pronouns in their posts more often.

Despite above differences, there were some findings in line with the previous literature. Present tenses and sadness words have also been identified as valid cues to recognize depression in prior research (28, 63). Depressed people have been described as “stucking in the past” (64), and thus focusing more on the past rather than the present. Therefore, a lower rate of present tenses words might manifest the possibility of depression. And the more frequent use of sadness words supported that depressed individuals expressed more negative emotions (53). Furthermore, achievement words were less mentioned by the depressed group in our study. O’Connor et al. (65) suggests that depressed people were significantly higher in survivor guilt. Survivor guilt refers to a dysfunctional belief possessed by individuals who believe that the pursuit and achievement of their own happiness and fulfillment will cause others to suffer by comparison. Therefore, we inferred that depressed patients were less likely to share their achievements on the posts.

#### 4.2.2. Chinese Suicide Dictionary

For suicide-related expressions, results indicated that psychache words, personality words and guilt/shame words could help identify people with depression. Psychache words (e.g., want to cry, loneliness) reflect one’s psychological distress, which are more likely to experience and express by people in depression (66). Guilt/shame words (e.g., lose status, make an apology) embody a sense of guilt and shame. It has been suggested that survivor guilt and omnipotent responsibility guilt were important factors in depression (65). Personality words reflect negative personality such as inferiority complex. The results showed that compared to healthy individuals, those with depressive tendencies instead less likely to mention negative personality words in their public discourse. This brought new insights into our understanding of how depressed people present their self-image on social media. Rosen et al. (67) illustrated that frequent impression management such as updating profile information was positively related to depression. Thus, depressed people might also be motivated to avoid negative self-disclosure in order to leave a better impression with others.

#### 4.2.3. Individualism/Collectivism Dictionary and Chinese Version of Moral Motivation Dictionary

Our study shows that the non-depressed group scored higher in collectivism and communion. In collectivist cultures, people stress the importance of the community and value the trait of altruism. Previous literature shows that collectivism could predict greater social support and enhance group identification (68, 69). Further, a higher level of social support and group identification could buffer vulnerable individuals from stressful conditions, and prevent people from developing depressive symptoms (70, 71).

Communion is the motive to promote the interests of others, with themes of caring for others and contributing to the society, involving qualities such as benevolence, attachment and empathy (72). Indeed, when we compare communion with collectivism values, it can be seen that communion could also be encouraged in the collectivism culture. A study conducted in Japan, also a typically collectivist country like China, found that communion was positively associated with social support, and contributed to psychological well-being (73). Thus, in East Asian societies, collectivism and communion could probably act as protective factors for depression.

#### 4.2.4. Chinese Version of Moral Foundation Dictionary

It can be told that the Chinese Version of Moral Foundation Dictionary provides a lot information for making prediction, as nearly half of the lexical features from this dictionary were selected by our model. Graham et al. (74) came up with five moral foundations rooted in human nature, and each foundation has positive and negative dimensions, which are care/harm, fairness/cheating, loyalty/betrayal, authority/subversion, and sanctity/degradation. The part before the slash is the name of a moral foundation (i.e., virtues), and the part after the slash refers to the corresponding foundation-violating behavior (i.e., vices). Results show that the depressed group varied the healthy group in terms of the use of fairness vice words, sanctity vice words, authority vice words, authority virtue words and morality general words.

The fairness foundation was based on ethic of justice, emphasizing on the rights and welfare of individuals. A higher score for the depressed group on fairness vice (cheating) represented that they mentioned more about the fairness-violating behavior (e.g., prejudice, inequality) than the normal group. We suggested that this group might perceive a lower level of social fairness, and thus resulting a poorer mental health status, especially for the disadvantaged minority group. It was expected that individuals who hold fewer favorable beliefs about the fairness toward society were less likely to believe their hard work and efforts would pay off, leading to the increase of emotional depression (75, 76).

Sanctity, which is also called purity, did not only focus on pathogen avoidance to protect us from being contaminated, but also emphasizing on cultivating a more spiritual mindset by living in a pure and sanctified way. Han et al. (77) found that purity vice had positive significant mediating effect on suicidal behavior *via* the mediator psychache. They pointed out this could be due to the fact that the psychology of purity was associated with stigma, which further has a negative impact on mental health.

The authority foundation focused on forging beneficial relationships in the hierarchy. This foundation was comprised of both virtues of subordinates (e.g., obedience and respect for authority) and virtues of authorities (e.g., leadership and protection). The authority virtue words included expressions that promotes obedience and leadership (e.g., obey, respect), whereas the authority vice words contained expressions that describes subversion (e.g., rebel, riot). We observed that non-depressed populations mentioned both the authority vice vocabulary and the authority vitue vocabulary more frequently than depressed populations, a phenomenon that seems to contradict each other. However, in our opinion, the greater use of these two vocabularies is indicative of the group’s emphasis on adherence to authority, since most of the words in the authority vice category are negative descriptions that are more likely to be used in the context of accusations and condemnations of violations of authority. The Chinese culture has been largely influenced by Confucianism, which emphasizes order and conformity to authority. For the non-depressed group, their obedience to authorities enhanced their adaptation to the social environment, reducing the likelihood of depression (78).

#### 4.2.5. The significance of those beyond basic expression features for depression identification

Our results not only demonstrated the importance of word frequencies of online expressions for depression recognition, but more importantly, brought new insights into the relationship between depressive symptoms and linguistic expression through building interpretable linear model. This study along with previous research using LIWC to detect depression have shown a tendency for depressed individuals to use several basic categories of words, including tenses, emotion, personal concerns and so on. Other features found to play an important role in our study, such as communion, focused on the socio-cultural elements of linguistic expression, and reflecting the motivation of users’ social behavior. This finding suggests that researchers should pay more attention on the socio-cultural features of linguistic expression while observing and monitoring depression. The reason why these features have not been explored in prior literature on depression recognition might be that researchers believed that these socio-cultural factors were relatively more difficult to reflect in the form of word frequencies. Our study, on the other hand, confirmed that the calculation of word frequencies could, to some extent, extract socio-cultural elements in linguistic expression. And these socio-cultural features had a non-negligible role in the identification and monitoring of depression.

To conclude, this study showed the feasibility of relying solely on lexical features of the text for depression detection, with all the five dictionaries contributing to the prediction to varying degrees. That is to say, in addition to LIWC/SCLIWC, which was frequently used in previous research, lexical features related to cultural psychology and suicide risk also played a role in the identification of depression. To better identify the mental health status of individuals on social media, we are supposed to attach more importance of the textual expressions related to culture psychology and suicide risk, as these factors could provide information on individuals’ predispositions shaped by the culture and also their perception toward the world.

### 4.3. Implications

The present study demonstrated that public discourse could reflect one’s mental status to some extent. Public discourse reveals important information about individuals’ different values and their perception toward the world (79, 80). Through linguistic analysis along with machine learning methods, we could discriminate depressed content from non-depressed content.

One of the advantages of this model is the applicability to text-only scenarios. A number of previous studies have utilized social media users’ social networking behaviors and profile information to train recognition models (30, 31, 50). However, if such personal information was not accessible, the validity of these models would be greatly compromised and even completely lose their predictive power. By contrast, our study only extracted features from text, which enables our model generalizable across platforms, especially those where only text can be collected.

Furthermore, to our knowledge, this is the first research that took cultural factors and suicide related expressions into account in feature extraction. This study deepens our understanding of the possible roles of cultural psychological related factors and suicide expression in depression identification, and shedding light on the relationship between these lexical features and depression.

### 4.4. Limitations

This study has a few limitations. Firstly, our analysis of the text is limited to the extraction of word frequency features, which have a very limited degree of representation of text information. The use of more different feature extraction strategies could enrich our feature set and perhaps further improve the performance of the model. Secondly, we have minimum requirements for posting frequency and total word count while selecting users to construct our dataset, so the model will be more suitable for predicting the depression status of individuals who are more active on social media. Thus, there might be a sampling bias, as some individuals with depression would not be willing to share their life on social platform, which would limit the generalization of our results and model to other populations. Thirdly, we only recruited some machine learning methods (e.g., random forest regression) to build the predictive model. In fact, in addition to regular machine learning methods, deep learning could also be utilized for depression detection, and previous studies have validated the effectiveness of this approach for depression prediction (81). Fourthly, the feature to sample ratio was suggested to be less than 0.10 to avoid the overfitting problem (82). In our study, the feature to sample ratio was 0.15, which is close but there is still room for further improvement. Thus, expanding the sample size to improve the robustness of the model is recommended for future research. Besides, future research could try more different combinations of texture feature extraction methods (e.g., n-gram, word2seq) and deep learning algorithms to further improve the model performance.

## 5. Conclusion

This study found that depression could be detected solely through word frequency features by machine learning methods. This model could have potential value in the screening for depression and be able to generalized across platforms. Furthermore, our study demonstrated that in addition to LIWC, which was commonly used in previous studies, lexicons related to cultural psychology and suicide risk were also associated with depression, and could contribute to the recognition of depression.

## Data availability statement

The raw data supporting the conclusions of this article will be made available by the authors, without undue reservation.

## Ethics statement

The studies involving human participants were reviewed and approved by the Scientific Research Ethics Committee of the Chinese Academy of Sciences Institute of Psychology. The patients/participants provided their written informed consent to participate in this study.

## Author contributions

SL, NZ, and XR contributed to the conception and design of the study and final version of the manuscript. SL was responsible for the data collection and the statistical analysis. SL and NZ wrote the manuscript. YD helped with the manuscript revision. All authors contributed to the article and approved the submitted version.
